# New Devitalized Freeze-Dried Human Umbilical Cord Amniotic Membrane as an Innovative Treatment of Ocular Surface Defects: Preclinical Results

**DOI:** 10.3390/jfb13030150

**Published:** 2022-09-13

**Authors:** Sophie Cognard, Laurence Barnouin, Justine Bosc, Florelle Gindraux, Marie-Claire Robin, Jean-Yves Douet, Gilles Thuret

**Affiliations:** 1 Clinique Vétérinaire des Ducs de Bourgogne, 11 Ter Rue Paul Langevin, 21300 Chenove, France; 2 Tissue Bank of France, 6 rue d’Italie, 69780 Mions, France; 3 Service de Chirurgie Orthopédique, Traumatologique et Plastique, CHU Besançon, F-25000 Besançon, France; 4 Laboratoire de Nanomédecine, Imagerie, Thérapeutique EA 4662, Université Bourgogne Franche-Comté, F-25000 Besançon, France; 5 Small Animal Clinic, Université de Toulouse, ENVT, 31300 Toulouse, France; 6 IHAP, Université de Toulouse, INRAE, ENVT, 31300 Toulouse, France; 7 Biology, Engineering and IMAGING of Corneal Graft, BiiGC, Faculty of Medicine, Health Innovation Campus, 10 rue de la Marandière, 42270 Saint-Priest en Jarez, France

**Keywords:** deep ocular defect, human umbilical cord amniotic membrane, placenta tissue, graft, sclera regeneration, corneal healing

## Abstract

A preclinical study was performed to investigate the efficacy and safety of a new viral inactivated, devitalized, freeze-dried and gamma-sterilized human umbilical cord amniotic membrane (*l*hUC-AM) for the treatment of deep scleral and corneal defects with or without perforation. Firstly, *l*hUC-AM was investigated on experimental deep sclerectomy in rabbit eyes (*n* = 12) and compared to autograft (*n* = 4) on cross section histology. Secondly, *l*hUC-AM was studied on a selected series of uncontrolled cases of corneal defects (*n* = 18) with or without perforation, in dogs and cats. *l*hUC-AM tolerance, reconstruction of the deep corneal lesion and recovery of the structural aspect of the tissue were followed post-surgery. In experimental deep sclerectomy, histology showed that the *l*hUC-AM was well tolerated and degraded completely in 45 days while allowing an overall quality and kinetic of scleral regeneration, similar to autograft. In the clinical situations, *l*hUC-AM was well tolerated, with ocular inflammatory signs quickly decreasing after surgery. Mean follow-up was 16.40 ± 11.43 months. In 15 out of 18 cases, *l*hUC-AM allowed ocular surface wound healing. The ocular surface was fully reconstructed three months after surgery. This study suggests a good safety and efficacy profile of *l*hUC-AM in the treatment of deep corneal or scleral defect in animals. This new tissue should now facilitate the treatment of severe ocular surface diseases in humans.

## 1. Introduction

Deep loss of substance in the sclera or cornea requires, most of the time, a rapid surgical management. The strategy choice for filling the defect depends on multiple factors: the type of defect (ulcer, keratectomy, degeneration and others), the possible superinfection of the defect or of the adjacent cornea, the possible perforation of the defect and the availability of substitute materials.

Several techniques/graft tissues have been described for the treatment of these ocular defects [1]. They include lamellar or penetrating keratoplasty [2,3,4,5], proved to be efficient with minimal complications but not always accessible or feasible in emergency. Corneoscleral/corneoconjunctival transposition [6,7]; conjunctival autografts, including island, pedicle, bulbar, bridge, or complete bulbar graft [1,8]; and autologous corneal graft [9], as well as cyanoacrylate tissue adhesive [10,11] have also been described. However, the corneal structure is partially restored and the transparency of the cornea is delayed for months or is impaired due to scarring. Trials with xenografts biomaterials were also realized with equine or bovine pericardium [12,13], porcine small intestinal submucosa [14,15,16], porcine urinary bladder [17,18] and bovine omentum [19] with promising results in animals. However, to the best of our knowledge, these biomaterials have never been widely distributed or commercialized for cornea repair, especially in humans.

Placental tissues, and especially the human amniotic membrane (hAM), have for several decades shown their numerous therapeutic advantages in both human [20] and veterinary [21] ophthalmology. Indeed, fresh, frozen or freeze-dried (lyophilized) hAM, thanks to its unique structure and myriad of physical and biological properties [22], has been used as an alternative treatment for ocular surface reconstruction after certain diseases, such as persistent epithelial defects and ulceration of the cornea or acute chemical burns [23,24,25,26]. Used alone or in association with conjunctival graft, hAM transplantation reduces inflammation and scarring, promotes epithelialization and wound healing and improves the management of pain [27]. It was shown to be an effective surgical technique with excellent functional and cosmetic outcomes for the treatment of both complicated corneal ulcers in dogs [28] and corneal sequestrum in cats [29,30]. As is the case with hAM, the human umbilical cord (hUC) also has unique biological properties related to its composition [31]. It consists of hUC amniotic membrane (hUC-AM), the outermost layer, which surrounds a mucoid connective tissue called “Wharton’s jelly”, and hUC blood vessels [32]. Wharton’s jelly is the extracellular matrix with high levels of collagen, hyaluronic acid and sulfated glycosaminoglycan (GAG) [33]. The hUC-AM is particularly resistant to twisting and shock. Its physical and biochemical characteristics make it of special interest as a scleral substitute or for deep corneal ulcers.

In the present work, we present the safety and efficacy preclinical study of a new viral inactivated, devitalized, freeze-dried, gamma-sterilized human umbilical cord amniotic membrane called *l*hUC-AM for severe ocular surface defects.

## 2. Materials and Methods

### 2.1. Tissues Preparation

The *l*hUC-AM (SclerFIX; Tissue Bank France, Mions, France) was prepared as follows: hUC tissue attached to placenta was collected individually from birth delivery after obtention of donor’s consent for tissue donation and associated, legally required, serological analyses (hepatitis B and C, human T-lymphotropic and human immunodeficiency viruses and syphilis). Placenta was transported in 0.9% sodium chloride solution at 4 °C. In a clean room, the hUC was washed with saline to remove blood clots. After immersion in sterile water between 2 and 72 h, the hUC was opened and the vessels were removed from the lining. The hUC-AM was then preserved in dry conditions at −80 °C until treatment for up to 24 months without biological changes. It was treated by a process consisting of viral inactivation chemical treatment, freeze-drying and gamma-sterilization allowing its conservation for 5 years at room temperature. This innovative patented process secured the product while maintaining its structural and biological integrity. The collection, the development of the process with animal studies was authorized by the French Research Ministry: number DC-2018-3200.

### 2.2. Histological Evaluation on Rabbits

#### 2.2.1. Animals

Rabbit experiments were carried out in accordance with European and National regulations for the protection of animal rights and with internal Charter on the Humane Care and Use of Laboratory Animals. The experimental design was approved by a local French Animal Care and Use Committee. Sixteen healthy male HY79b pigmented rabbits aged over 8 weeks were housed under controlled conditions of temperature and light cycle (12 h light:12 h dark) in an animal housing facility with ad libitum access to food and water. Rabbits were acclimatized for 7 days before surgery.

#### 2.2.2. Surgical Procedure

A deep sclerectomy was performed on the left eye of 16 rabbits under an operating microscope. Briefly, the dorsal bulbar conjunctiva was incised through the conjunctival sac to expose the sclera. A 4 × 4mm square scleral incision was made 3 mm posterior to the limbus. Dissection was continued until reaching the choroid. The defect thus created was filled either by a 4 × 4 mm square of *l*hUC-AM with epithelial side facing up (*n* = 12) or by replacing the freshly excised sclera (*n* = 4). Grafts were sutured using 8 to 10 separated stitches of 9/0 PGA filament (Vicryl^®^, Ethicon, Johnson and Johnson, Issy les Moulineaux, France). Bulbar conjunctival incision was then closed with continuous suture, using 6-0 PGA polyfilament (Vicryl^®^, Ethicon, Johnson and Johnson, Issy les Moulineaux, France).

#### 2.2.3. Post-Operative Follow-Up and Evaluations

Daily clinical follow-up was performed, and a thorough ophthalmologic examination was performed at 3, 7, 10, 14, 21, 30 and 45 days (D) after surgery to search for: blepharospasm, epiphora, conjunctival hyperemia, conjunctival oedema, iritis. Intraocular pressure (IOP) was monitored by rebound tonometry (Icare TONOVET Plus, Icare Finland Oy, Vantaa, Finland) daily and at a fixed time for 45 days.

At D7, D14, D30 and D45, three animals from the SclerFIX group and one from the autologous group were sacrificed to perform a histopathological study of the eyeballs. The two eyeballs were removed. After transconjunctival enucleation, the globes were fixed with formalin (immersion of formalin 4% then injection via optic nerve after 24 h of fixation) before macroscopic analysis. Macroscopic analysis focused on tissue reactions at the site of implantation: hematoma/bleeding, inflammatory exudate, fibrous reaction, tissue necrosis, as well as the presence, form and location of the graft and the presence of possible residues resulting from its degradation. A macroscopic assessment of the intraocular structures was performed after the eyeballs were cut in half. 

The eyeballs were then embedded in paraffin blocks. Sections at a thickness of 3–4 μm were made from the paraffin blocks (1 section/slide, 1 slide/eye). Sections were stained with hematoxylin-eosin for histopathological analysis. Red Sirius staining was performed for evaluation of collagen fibers deposits. The slides were read by a pathologist who was not aware of the treatment group. A semi-quantitative grading of local tissue tolerance and integration of the biomaterial was carried out using an intensity score ranging from 0: absent to 5: severely present. It focused on the following parameters: inflammation, oedema and congestion, necrosis, fibrosis within and around scleral implantation site, reduction in the wide of the defect and residues of biomaterial. Scleral reconstruction was evaluated according to scleral defect filling, subconjunctival space and bleb formation, as well as the quality of the reconstructed tissue. Any other observation was also recorded.

### 2.3. Clinical Evaluation on Cats and Dogs

#### 2.3.1. Animals

Animals were recruited during consultations in a veterinary clinic in France. Participation to the study and treatment with *l*hUC-AM was suggested for dogs and cats presenting with deep ocular lesions reaching the posterior third of the cornea with a risk of perforation. These lesions could be ulcer or excision of sequestrum after keratectomy. Animals were included in the study after informed written consent of the owner.

#### 2.3.2. Surgical Procedure

Surgery was performed under general anesthesia in the veterinary clinic. The corneal area to be grafted was prepared after standard surgical cleaning. In the case of feline sequestrum or non-perforating ulcer surgery, the necrotic epithelial-stromal area was marked for keratectomy with a calibrated trephine of diameter 0.5 mm greater than the lesion. A lamellar dissection with a crescent knife was discovered to obtain a flat, smooth and calibrated keratectomy bed. In perforated ulcer surgeries, the ulcer margins were resected with corneal scissors to obtain a healthy peripheral collagenous recipient zone. The *l*hUC-AM was trephined or manually cut to fit the shape and size of the defect. It was placed at the bottom of the defect before being rehydrated with a few drops of 0.9% sodium chloride solution. The graft was then sutured using 3 mm separated stitches of 9/0 PGA filament (Vicryl^®^, Ethicon, Johnson and Johnson, Issy les Moulineaux, France) to the deepest part of the graft bed to ensure the good coaptation of the graft with the deep stroma.

A second layer of *l*hUC-AM was placed on top of the first one with stitches securing the two layers of *l*hUC-AM and the stromal margins when the lesion was very deep or perforating. Alternatively, a thin conjunctival pedicle graft of the same diameter as the lhUC-AM was used in addition to a monolayer of *l*hUC-AM when the perforation or loss of substance was larger than 8 mm or when the risk of aqueous humor leakage or superinfection was deemed high. The conjunctival graft was placed on top of the *l*hUC-AM for 45 days before being removed under local anesthesia. In this case, simple resorbable stitches secured the edge of the conjunctival graft, the edge of the *l*hUC-AM and the superficial stromal margin. Finally, a layer of hAM (VISIO-AMTRIX; Tissue Bank France) was placed on top of the *l*hUC-AM with 6 conjunctival resorbable stitches (9/0 Vicryl, Ethicon) when it lacked continuity with the epithelial plane. The nictitating membrane was closed for 15 days in all animals that did not receive conjunctival pedicle graft.

#### 2.3.3. Post-Operative Follow-Up and Evaluations

Animals were kept under observation for 24 h. Then, discharge and discomfort were monitored by the owner, with 2 veterinarian visits: one at Day 5 and one at Day 15, which allows opening of the nictitating membrane. 

The recommended initial follow-up was at least 3 months post-surgery. However, controls depended on the evolution of each case. Follow-up visits were regrouped by time period post-surgery: between 1 and 14 days, between 15 and 29 days, between 30 and 89 days and 90 days after surgery.

The reconstruction of deep corneal lesions and recovery of the structural aspect of the corneal tissue was assessed by searching for the persistence of the ulcer post-surgery (yes/no), the reconstruction of the cornea at level of the surface (yes/no), the epithelialization (partial/total), the pigmentation (yes/no) and the transparency (grade 0 = very few infiltrates, grade 1 = few infiltrates, grade 2 = significant infiltrates and grade 3 = major infiltrates or opacity). In the case of cats, ocular surface healing was also followed by optical coherence tomography (OCT) when the animal was manageable without anesthesia.

The evolution of the inflammatory criteria was evaluated between the inclusion visit and the post-surgical visits by the following ocular symptoms: corneal edema and congestion (from 0 = absence to 4 = diffuse), blepharospasm (yes/no), watering and discharge (from 0 = absence to 4 = foul smelling constant discharge). Vascularization was scored from 0 (absence) to 4 (4 quadrants) and evaluated in comparison with first post-surgical visit.

Feeling of veterinarian and owner about the recovery were also recorded at each follow-up visit.

Safety was followed by the recording of adverse events (AEs) by the veterinarian throughout the whole duration of the study.

### 2.4. Data and Statistical Analysis

For clinical evaluation, animals followed less than 45 days were excluded from the analysis. For histological and clinical evaluation, normality of data distribution was analyzed with a Shapiro–Wilk test. When the variable followed a normal distribution, a Student *t*-test was used to compare measurements between groups, if not a Mann–Whitney U test was used. Rejection of the null hypothesis was defined as α < 0.05 (two-tailed). Statistical analyses were performed using R 4.0.2 (R Core Team, Vienna, Austria).

## 3. Results

### 3.1. Histological Evaluation on Rabbits

#### 3.1.1. Clinical Results

**Figure 1:**
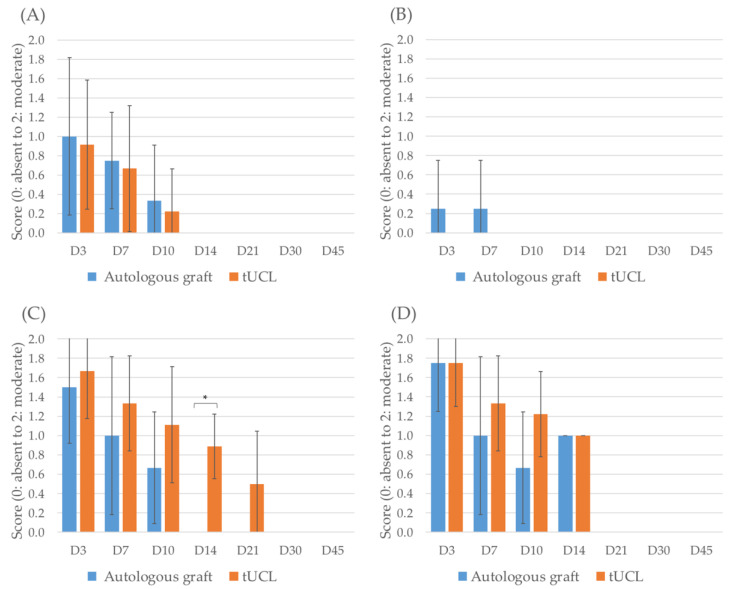
Monitoring of post-operative blepharospasm (**A**), epiphora (**B**), conjunctival hyperemia (**C**) and conjunctival oedema (**D**). The asterisk indicates a significant difference between the autologous graft and the tUCL groups.

No iritis, Tyndall effect or variation of the IOP were observed in either group during the study. Blepharospasm, epiphora, conjunctival hyperemia and conjunctival oedema progressively decreased after surgery and disappeared in both groups after 30 days (Figure 1). At 14 days, conjunctival hyperemia was observed in all but one animal treated by *l*hUC-AM at weak intensity while in none of the animals treated with autologous graft at 14 days (*p* = 0.009). No significant differences in blepharospasm, epiphora and conjunctival edema were observed between animals treated by *l*hUC-AM product and the animal treated with the autologous graft.

#### 3.1.2. Local Tolerance and Biodegradability of the lhUC-AM

**Figure 2:**
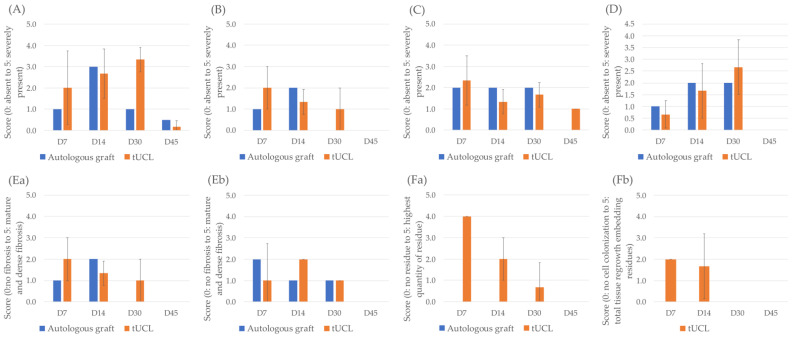
Monitoring of local tolerance and biodegradability of the implanted biomaterial: inflammation (**A**), congestion (**B**), oedema (**C**), necrosis (**D**), fibrosis around (**Ea**) and in (**Eb**) the biomaterial, amount (**Fa**) and colonization (**Fb**) of biomaterial residue.

Inflammation was greater with *l*hUC-AM implantation at 7 days and 30 days but decreased drastically afterward and became minimal and at the same level as animals with autograft at 45 days after surgery. Moreover, animals implanted with *l*hUC-AM showed congestion and oedema of the same intensity as other animals but over a longer duration. The presence of necrotic tissue was similar between the two groups. After 45 days, low-intensity oedema persisted in animals treated by *l*hUC-AM but no necrosis or congestion was detected in either condition (Figure 2A–D).

Fibrosis was similar in the two conditions: it was mild to moderate and became a dense collagenous tissue with a parallel alignment at 30 days. No fibrosis was detected at 45 days either around or at the implanted site, for both autograft and *l*hUC-AM (Figure 2E).

The biodegradability of the implant was evaluated according to the amount of residue of the *l*hUC-AM. The biomaterial was observed within tissue at 7 and 14 days and appeared as an acellular fibrillar collagen membrane. At 30 days, some small and dense fibrous islands were present in the scleral defect, but it was not possible to discriminate degraded *l*hUC-AM from newly formed fibrous tissue. At D45, *l*hUC-AM residues were no more detected within the scleral (Figure 2F).

#### 3.1.3. Reconstruction of Scleral Defect

**Figure 3:**
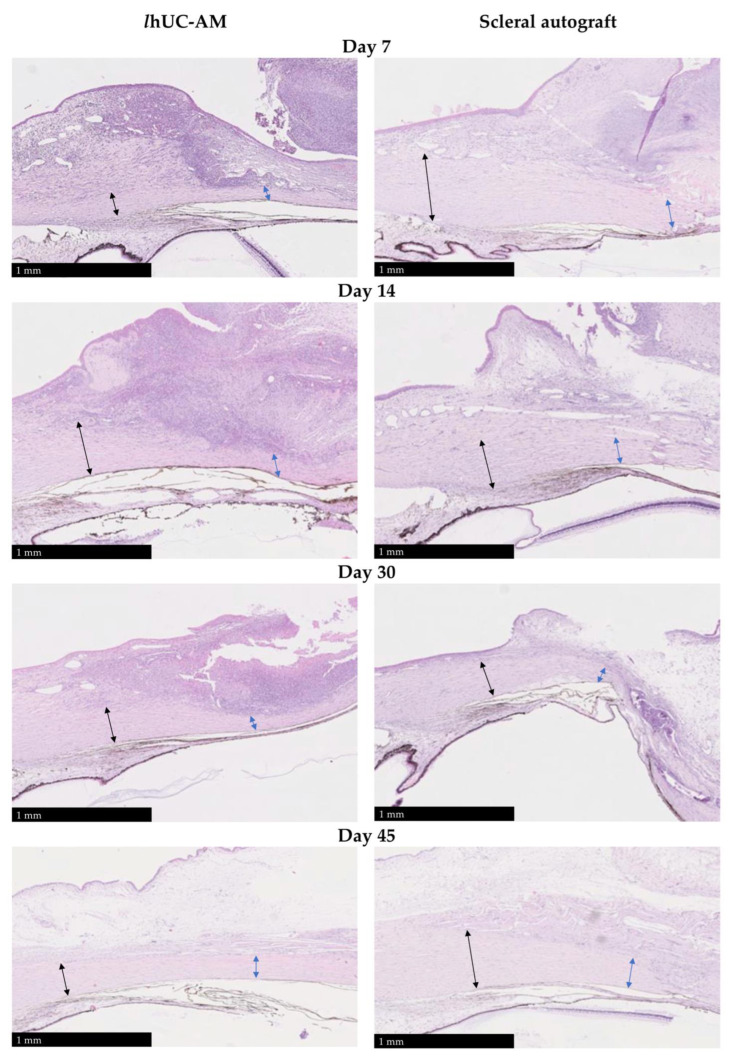
Kinetic of sclera reconstruction at day 7 (D7), D14, D30 and D45 in rabbits with or without *l*hUC-AM versus scleral autograft. Black arrows showed the normal sclera and blue arrows showed the thickness of the sclera at the site of the deep sclerectomy.

The filling of the defect was similar between animals with autograft and those with *l*hUC-AM with total filling after 45 days. Although a small amount of bleb was present for animals implanted with *l*hUC-AM after 30 days, the subconjunctival space was at a physiologic level at 45 days after surgery. The reconstruction kinetics were similar between autograft and *l*hUC-AM implantation (Figure 3). Finally, the quality of the reconstructed tissue in terms of collagen fibers and conjunctival epithelium was identical to the adjacent tissue at 45 days.

### 3.2. Clinical Evaluation on Cats and Dogs

#### 3.2.1. Animals

A total of eight dogs and nine cats, including one with bilateral affection, were included in the study (18 eyes) (Table 1). Mean age was 4 ± 3.5 years for dogs and 5 ± 5 years for cats. All dogs had a deep ulcer. For cats, two had a deep ulcer, one had a very large keratomalacia with a corneal perforation, one had a perforation following spontaneous expulsion of sequestrum, and six had a deep keratectomy after surgical removal of feline sequestrum. At inclusion, ulcer size was greater than 2 mm in 94% (17/18) of cases and greater than 5 mm in 61% (11/18) of cases. All animals showed clinical signs of severity (Table 1).

#### 3.2.2. Surgical Procedure

During surgery, six eyes received one-layer of *l*hUC-AM (33%, 4 cats, 1 dog). Double-layer of *l*hUC-AM was used in three animals (17%, 2 cats, 1 dog). *l*hUC-AM was combined with conjunctival pedicle graft in four animals (22%, 4 dogs) and with hAM in five animals (28%, 3 cats, 2 dogs).

#### 3.2.3. Follow-Up/Outcomes

Seventeen animals (18 eyes) were included in the study, three animals ended the study prematurely at days 2, 12 and 21 post-surgery due to failure and one case was followed up for only 45 days. Thus, 13 animals (14 eyes) were followed for at least 3 months. So, the mean follow-up was 16.40 ± 11.43 months (range, 1.5–36.5).

The success rate was 83% (15/18). The three cases of failure were: 1/a dog treated by *l*hUC-AM and conjunctival pedicle graft for deep ulcer presented a perforation with dehiscence of the *l*hUC-AM and conjunctival layers, leading to enucleation at day 2 post-surgery; 2/a dog treated by *l*hUC-AM + hAM for deep ulcer presented with a descemetocele with graft failure at 12 days post-surgery; 3/a cat treated by one-layer of *l*hUC-AM for deep ulcer had doubtful engraftment on the deep bed at 15 days post-surgery followed by mutilation and expulsion of the graft at 21 days.

**Figure 4:**
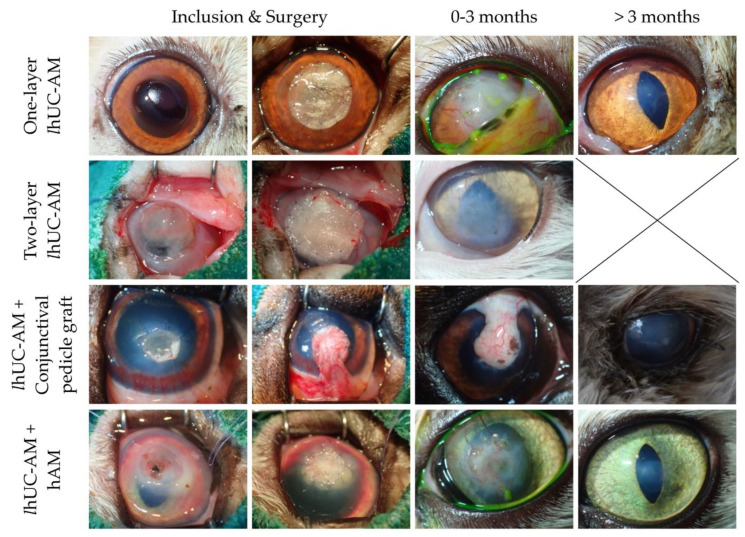
Follow-up of cats with deep corneal defects and treated by one-layer *l*hUC-AM, two-layer *l*hUC-AM, *l*hUC-AM and conjunctival pedicle graft or *l*hUC-AM and hAM.

There was no recurrence of the disease in our follow-up period (3–15 months). Photographs of the cornea of one case in each treatment group taken at inclusion, at surgery, and during follow-up are displayed in Figure 4. After 3 months post-surgery, all deep lesions were resorbed with reconstruction of the cornea at the level of the surface. Thus, there was no difference in term of resorption between groups treated by one-layer *l*hUC-AM, two-layer *l*hUC-AM, *l*hUC-AM and conjunctival pedicle graft and *l*hUC-AM and hAM (*p* = 1). All groups combined, total epithelialization was observed in 67% of the cases followed up at 15 days, 86% of the cases followed up at 1 month, then 100% of the cases followed up over one month. No corneal pigmentation was observed in the first 3 months following the surgery. However, corneal pigmentation was observed in two dogs (14%) treated by *l*hUC-AM and conjunctival pedicle graft, including one with glaucoma, during the long-term follow-up (>3 months). Grade 0 of transparency was observed in 90% of the cases at 3 months (9/10) and in 93% of the cases at more than 3 months (13/14).

All groups combined, ocular inflammatory signs quickly decreased after surgery. Corneal edema, blepharospasm and lacrimation decreased significantly a few days post-surgery. After 3 months, no animal had edema or blepharospasm, and three dogs (17%, one dog treated with by one-layer *l*hUC-AM, two dogs treated by *l*hUC-AM and conjunctival pedicle graft) still showed epiphora. Congestion also decreased significantly from 15 days post-surgery. After 3 months, only one dog had congestion and congestion was linked to glaucoma. In all groups combined, corneal vascularity decreased significantly between the first postsurgical visit and the visit beyond 3 months. The veterinarian and owner felt that all cases, excluding cases of failure and one case with glaucoma (78%), had significative improvement at more than 3 months, as compared to inclusion. The difference between inclusion and the long-term follow-up visit was still judged as better by both the veterinarian and the owner for the dog with glaucoma.

## 4. Discussion

Deep ocular surface defects are ophthalmological emergencies for the preservation of the vision and the integrity of the globe. Several grafting techniques, with varying efficacy and limitations, have been described and are emerging for the treatment of these types of defects.

*l*hUC-AM used in this study have the particularity to result from an innovative process, including chemical devitalization, viral inactivation, freeze-drying and gamma-sterilization. To the best of our knowledge, no published study has evaluated the use of a devitalized freeze-dried hUC-AM product for the treatment of these ocular conditions.

Thanks to this process and the adequate screening of the placenta donors, this type of product allows the use of secure grafts with no risk of transmitting bacterial, viral or fungal infections to recipients. Moreover, this *l*hUC-AM product provides additional advantages, such as high availability (thanks to the relative availability of placentas), long-term storage and shipment at room temperature. It can also be easily dimensioned at the size of the defect and sutured with less risk of tearing (unlike sometimes hAM). Composed of hAM and Wharton’s jelly, the hUC wall benefits from growth factors, hyaluronic acid and GAG [31,33], which could be a promising source for corneal surface regeneration.

In this article, we presented two investigations, one performed on rabbits, allowing histological evaluation, and one performed under routine conditions (veterinary clinic) allowing clinical evaluation. The histological evaluation following deep sclerectomy surgical procedure on rabbits showed that *l*hUC-AM is well tolerated by the recipient and quickly degraded while offering an overall quality and kinetic of scleral reconstruction similar to autograft. The newly reconstructed sclera has similar characteristics with the physiological sclera with a good tolerance. *l*hUC-AM, thus, happens to be as efficient as an autologous graft for transplant defect, which make it a good candidate for scleral transplantation.

The clinical evaluation on selected corneal disease in a small series of dogs and cats showed that the use of *l*hUC-AM graft appeared to be effective with good results in terms of corneal healing when used alone for treatment of deep ocular surface defects or when used in combination with additional superficial sheet in the cases of very deep (loss of substance larger than 8 mm), perforating defects or defects with high risks of complications, with total reconstruction of the cornea at the level of the surface 3 months post-surgery regardless the group. Healing kinetic was similar between the groups. The product also showed good tolerance with ocular inflammatory signs quickly decreasing after surgery and the improvement of transparency over time.

The three cases of the failure of this experiment were not related to the *l*hUC-AM product. The dog with failure due to glaucoma was a Shih Tzu, which is a breed with high risk of secondary hypertensive uveitis. The second dog was an English bulldog with wide central descemetocele, which are known factors of ulcer complications in dogs. The last failure was due to cat scratching self-mutilation after opening of nictitating membrane.

This clinical evaluation presents some limitations. Firstly, the *l*hUC-AM product has not been compared to a standard surgery. So, we cannot assure the superiority of this product over the other treatments presented in the introduction of this article [1,6,7,8,9,10,11,12,13,14,15,16,17,18,19].

But the filling capability or kinetic of regeneration of deep defects is an interest of *l*hUC-AM. Despite multilayer treatment, the risk of incomplete filling of hAM can impact visual results. Less filling after the excision of conjunctival tumor can generate a risk of perforation or fibrosis. The kinetic of regeneration of this *l*hUC-AM could solve the timeline of proton therapy in case of malignant tumor.

Secondly, the clinical evaluation was carried out with a small sample of animals and would deserve to be carried out on a larger scale.

However, in view of the good tolerance (histologically and clinically evaluate) and the good long-term prognosis provided by the *l*hUC-AM, it could be clinical tested in humans for patients presenting a large defect of the ocular surface or risk of scleral thinning after surgical excision of conjunctival tumor.

## 5. Conclusions

This preclinical study suggests a good safety and efficacy profile of freeze-dried hUC-AM in the treatment of deep corneal or scleral defect in animals. This new tissue should now facilitate the treatment of severe ocular surface diseases in humans.

## Figures and Tables

**Table 1:** Signalment, surgical details and outcomes of the treatment with *l*hUC-AM.

Case No.	Animal	Age	Indication	Defect Size	Treatment	Success	Follow-Up Time (Months)	Observations on Long Term (>3 Months)
1	Cat	11 years	Deep ulcer	>5 mm	2-layer *l*hUC-AM	Yes	1.5	-
2	Cat	6 years	Perforation after spontaneous expulsion of sequestrum	>5 mm	2-layer *l*hUC-AM	Yes	36	-
3	Dog	11 years	Deep ulcer	>5 mm	*l*hUC-AM +conjunctival graft	Yes	9	Corneal pigmentation, opacity, epiphora and congestion in two quadrants probably related to glaucoma
4	Dog	7 months	Deep ulcer	>2 mm	*l*hUC-AM + hAM	Yes	36.5	
5	Dog	3 years	Deep ulcer	>2 mm	*l*hUC-AM + hAM	No	-	Descemetocele with graft failure at 12 days post-surgery
6	Cat	7 years	Deep keratectomy after surgical removal of feline sequestrum	>5 mm	*l*hUC-AM + hAM	Yes	4	-
7	Cat	11 months	Deep keratectomy after surgical removal of feline sequestrum	>5 mm	*l*hUC-AM + hAM	Yes	14.5	-
8	Dog	11 months	Deep ulcer	>2 mm	*l*hUC-AM +conjunctival graft	Yes	31	-
9	Dog	14 years	Deep ulcer	>1 mm	2-layer *l*hUC-AM	Yes	16	-
10	Dog	3 years	Deep ulcer	>2 mm	*l*hUC-AM +conjunctival graft	Yes	16	Corneal pigmentation, epiphora
11a	Cat	6 months	Deep keratectomy after surgical removal of feline sequestrum	>5 mm	1-layer *l*hUC-AM	Yes	8	-
11b		9 months	Deep keratectomy after surgical removal of feline sequestrum	>5 mm	1-layer *l*hUC-AM	Yes	4	-
12	Dog	7 years	Deep ulcer	>5 mm	*l*hUC-AM +conjunctival graft	No	-	Perforation with dehiscence of the lhUC-AM and conjunctival layers, leading to enucleation at day 2 post-surgery
13	Cat	5 years	Deep keratectomy after surgical removal of feline sequestrum	>5 mm	1-layers *l*hUC-AM	Yes	14	-
14	Cat	20 months	Deep ulcer	>2 mm	1-layers *l*hUC-AM	No	-	Doubtful engraftment on the deep bed at 15 days post-surgery followed by mutilation and expulsion of the graft at 21 days
15	Dog	18 months	Deep ulcer	>2 mm	1-layers *l*hUC-AM	Yes	27.5	Epiphora
16	Cat	16 months	Very large keratomalacia with corneal perforation	>5 mm	*l*hUC-AM + hAM	Yes	11	-
17	Cat	6 years	Deep keratectomy after surgical rcat treated by one-layer oemoval of feline sequestrum	>5 mm	2-layers *l*hUC-AM	Yes	18	-

## Data Availability

The datasets used and/or analyzed during the current study are available from the corresponding author on reasonable request.
